# Misalignment of Reward Response With Healthful Behavior: An Underappreciated Driver of Population Health Deficits and Health Disparities?

**DOI:** 10.3389/ijph.2022.1604830

**Published:** 2022-09-15

**Authors:** R. Scott Braithwaite, Mark D. Schwartz

**Affiliations:** Grossman School of Medicine, New York University, New York, NY, United States

**Keywords:** public health, population health, hedonic reward system, socioeconomic status, addiction science, evolution, alcohol use disorders, substance use disorders

## Abstract

Socioeconomic status-related (SES-related) health disparities are worsening across resource-rich environments, despite increased knowledge about health determinants and inducements for healthful behavior change. We ask whether insights from addiction science and evolutionary biology may assist understanding and counteracting SES-related health disparities. It is known that a mismatch between evolved traits and behaviors that conserve energy drives many health deficits. We posit that this energy mismatch is one manifestation of a more expansive mismatch in levels of reward activation, between environments more versus less manipulated by human activity. This larger mismatch explains why SES-related health disparities arise not only from overeating and excessive sedentism, but also from alcohol, nicotine, other substances, and mood disorders. Lower SES persons are more likely to have lower baseline reward activation, which leads to higher prioritization of reward elevating activities, and at the same time are less likely to act on knowledge about unhealthfulness of behaviors.

## Introduction

Population health deficits and socioeconomic status (SES)-related health disparities are worsening in the U.S. and in other resource-rich environments. The United States could gain an additional 5.9 years of life expectancy (from 78.8 years to 84.7 years) if these population health deficits were eliminated [1]. Of this 5.9 years shortfall, physical inactivity (1.5 years), unhealthy diet (1.1 years), and smoking (0.5 years) are especially notable contributors. Unhealthy alcohol use and substance use have further fueled increases in SES-related life expectancy gaps. We posit that greater success in counteracting these trends may require a broader perspective than is routinely employed in population health or public health [2]. This perspective would draw on findings from the addiction and evolutionary sciences, and would consider how resource-rich environments offer a plentitude of hedonic rewards that are novel from an evolutionary perspective and are sufficiently potent to induce addiction-like changes in brain reward circuitry. These changes favor behaviors, such as overeating, sedentary lifestyles, and substance use that activate the reward system, even when these behaviors are known to be unhealthy.

## Reward System Mismatches Are Ubiquitous in Resource-Rich Societies

Individuals in reproducing populations must survive until reproductive age and must successfully reproduce to pass on their genes to the next generation. To survive and successfully reproduce, individuals must procure energy, conserve energy whenever feasible, avoid trauma and illness, mate, and reproduce. When energy sources are scarce, and trauma avoidance and reproductive opportunities are uncertain, achieving these goals requires reinforcement by a potent reward mechanism together with a tolerance for risk. In the case of humans in a context of scarcity, uncertainty, and danger, metabolic and reward system responses promote a “faster” versus a “slower” lifestyle [3, 4], involving obtaining energy, such as eating calorically dense food; conserving energy, being physical inactive, and pursuing reproductive opportunities. A tolerance for risk is essential because many necessary activities are uncertain and involve added exposure to harm.

However, in resource-rich environments, plenty, certainty, and safety have replaced scarcity, danger, and uncertainty. Obtaining and conserving energy no longer requires eating as much calorically dense food as possible or avoiding unnecessary physical exertion. Obesity no longer puts humans at greater risk of predation. Yet Individuals still experience reward response from obtaining and conserving energy, due to the persistence of evolutionarily favorable “thrifty genotypes” (i.e., genetic disposition to be metabolically efficient), and “thrifty phenotypes” (i.e., phenotype of metabolic efficiency induced by fetal scarcity). Further, people survive longer in resource-rich environments, which magnifies the adverse health impacts of energy excess, because myocardial infarctions, strokes, diabetes-related complications, and obesity-related cancers occur disproportionately after reproductive age.

It is well established that a mismatch between energy needed and energy consumed in resource-rich environments leads to population health deficits. However, it is not generally appreciated that this energy mismatch is only one among many mismatches in reward system activation that resource-rich environments have enabled, between activation episodes that are adaptive from an evolutionary perspective and activation episodes that are evolutionarily novel, and often lead to unhealthful and maladaptive consequences [2]. This larger mismatch may explain why major population health deficits arise not only from overeating and physical inactivity but also from other volitional activities that overdrive the reward system by perturbing its set point or by forcing repeated excursions beyond its usual operating range. These unhealthful behaviors include major drivers of population health deficits such as smoking, unhealthy alcohol use, substance use, sedentary behavior, unhealthy diets, and obesity-related illnesses [2], as well as more minor drivers such as beta-endorphin-mediated ultraviolet radiation-seeking behavior (i.e., compulsive tanning) [5], excessive internet use and/or gaming disorder [6], exercise addiction [7], problem gambling [8], and binge behaviors underlying other “process addictions” [2].

As an example of the commonalities underlying these seemingly diverse phenomena, severe obesity [9], problem gambling [8], exercise addiction [7], and internet gaming disorder are characterized by changes in brain reward circuitry that lead to adaptation and tolerance, and are similar to those in substance abuse. In particular, the mesolimbic dopaminergic reward circuit displays dopamine receptor downregulation, lower dopamine levels, and blunted dopaminergic response to stimulation, yet with neural hyper-responsiveness to anticipatory cues. Other brain circuits (e.g., prefrontal cortex) and neurotransmitter systems (e.g., the mu-opioid receptor) are also part of this network of reward-related adaptations [9], and the recruitment of these circuits is modulated by chronic stress through projections from the hypothalamic-pituitary-adrenal (HPA) axis.

## Reward System Mismatches Depress Population Health and Augment SES-Related Health Disparities

**FIGURE 1:**
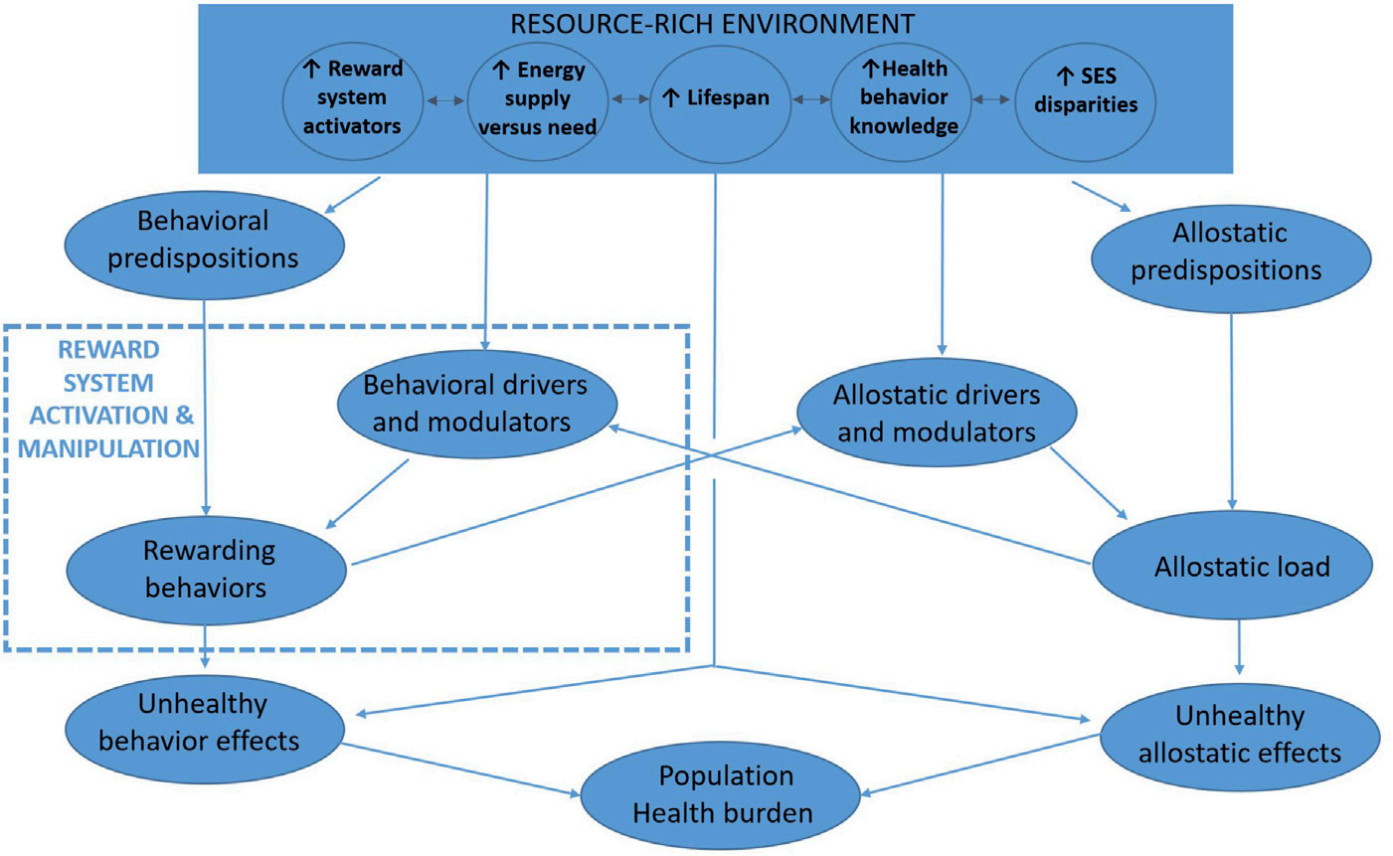
Conceptual model of reward activation and population health in resource-rich environments (New York City, U.S.A. 2022). Note: Resource-rich environments have brought evolutionarily rapid changes impacting the difference between availability and needs of metabolic energy, plentitude and potency of other reward system activators, lifespan duration, health behavior knowledge, and socioeconomic status gradients. Because many reward-activating behaviors are unhealthful and self-perpetuate through addiction-like mechanisms, and because increasing lifespan together with increasing health knowledge has unveiled their unhealthful effects, substantial preventable morbidity and mortality is known to occur from downstream conditions including obesity, diabetes, smoking, unhealthy alcohol use, substance use, chronic pain, and mood disorders. Lower socioeconomic status persons may disproportionately incur these health effects because their higher allostatic loads sensitize the appeal of reward system activation while blunting the salience of remote and uncertain unhealthful consequences. Individual behavioral predispositions (e.g., genetic susceptibility to alcohol misuse) and environmental modulators (e.g., availability and behavioral normativity of consuming alcohol) impact the likelihood that reward-seeking behaviors will occur (e.g., unhealthy alcohol use) and will consequently lead to poor health (e.g., liver disease). Analogously, individual allostatic predispositions (e.g., low stress resilience and/or high impulsivity) and environmental modulators (e.g., low social support) impact the likelihood that an allostatic load (e.g., poverty) will lead to poorer health (e.g., cardiovascular disease). The allostatic and behavioral pathways reinforce one another, yielding the unhealthful behaviors that mediate approximately half of socioeconomic status-related health disparities.

Figure 1 depicts a way of understanding the interrelatedness of reward activation and population health in resource-rich environments and how that relation is amplified by low SES at a population-level. Resource rich environments provide more potent, plentiful, and sometimes harmful options for reward activation. Indeed, resource-rich environments often occur with, and perhaps because of, economic systems that provide goods and services that are desired, regardless of the whether the desire pathologically overactivates the reward system or causes harmful health effects.

At the same time, resource-rich environments enable longer lifespans over which these harms can manifest, for multiple reasons including improved societal health knowledge (e.g., understanding of infectious diseases) and mitigation of external forces of mortality (e.g., lower risks of starvation and exposure). However, resource-rich environments encompass a wide spectrum of SES gradients, and higher SES gradients exact their own stressors.

Allostatic load is [10] the global physiological “wear and tear” resulting from adaptation to psychosocial adversities in the environment through stress response systems. Drivers of allostatic load are more common among lower SES persons, and include abuse, discrimination, low education or other agency impairments, poverty or attendant insecurity about obtaining necessities (housing, food, etc.), trauma, low social support, and stigma. The physiological impact of allostatic drivers may be buffered or accentuated by individual modulators or predispositions such as stress resilience.

Higher allostatic load leads to physiologic dysregulations that lower baseline reward activation, including chronic activation of the HPA stress circuit with correspondingly elevated cortisol levels, greater autonomic nervous system reactivity, and a pro-inflammatory and immunomodulatory milieu that accelerates vascular disease. While interactions between the HPA stress circuit and the mesolimbic reward circuit normally align reward response with activities necessary for survival and reproduction, in the setting of chronic stress there is maladaptation. Persistent HPA activation downregulates the brain’s reward system circuitry and heightens sensitivity to activating stimuli such as hypercaloric food or substances of abuse. Chronic HPA activation also reduces resilience to subsequent stressors as well as inducing and reinforcing neurocognitive changes and mood disorders [10]. Heightened sensitivity to reward activators in the presence of allostasis is seen in population-based and clinical studies, which indicate a strong relationship between number of severe stressful life events, obesity, and addiction vulnerability.

Experimental data on the role of allostasis on behavior is necessarily limited to animal studies, but their results are compelling and are consistent with observations in humans. Animals experimentally exposed to social stress stimuli, such as predator scent or social dominance challenges, consume more calories, self-administer more cocaine, methamphetamine, alcohol, and opioids compared to control animals who are not randomized to social stress exposure [11, 12] and exhibit anxiety and depressive phenotypes [12]. Further, animal experiments support the commonality of pathways invoked in reward response aberrations across a wide range of rewarding stimuli. For example, food-bingeing animals that are food-restricted opt to consume more alcohol.

### Interactions Between Allostatic Load and Rewarding Behaviors

Higher allostatic load amplifies the appetitive nature of reward activation and leads to more frequent and intensive rewarding behaviors, which are sometimes unhealthful. The disposition to seek reward may be buffered or accentuated by individual characteristics such as impulsivity, conscientiousness [13], perceived behavioral control, expectations regarding appetitive and aversive responses, and genetic predisposition to addiction, as well as by social and environmental factors such as a behavior’s social desirability, its normative status, and the admixture of prevalent facilitators and barriers (e.g., greenspace, food deserts). Rewarding behaviors resist change not only by providing immediate reward activation and but also by inducing brain adaptations that require greater “doses” of unhealthy activities to achieve the same level of reward. Behavior-related harm may be counteracted by increased societal knowledge about the unhealthful consequences of behaviors. However, this knowledge is sometimes less actionable for low SES persons because their baseline reward system activation is more likely to be depressed, heightening the appetitive lure of activating stimuli.

Additionally, while it is common for people to value current benefits or harms more than future benefits or harms (i.e., time preference or “delay discounting”), the differential valuation of current compared to future benefits or harms is greater in lower SES persons yielding decision-relevant timeframes that end before the delayed harms of unhealthful behaviors manifest. The role of time preference in facilitating unhealthful behavior is particularly strong for overeating and smoking [14]. Low SES persons may also lack the monetary and social capital (e.g., social support and health supporting institutions) to pursue healthful behavior change particularly when the healthful behavior is non-normative in their cultural contexts. Lower SES communities, even in otherwise resource-rich environments, have less access to high quality food sources, walkable areas, and recreational or exercise facilities. Finally, while health systems may sometimes blunt the unhealthful effects of harmful behaviors, low SES persons may access health systems often or less effectively because of financial barriers, mistrust, navigation challenges, lower quality, and lack of concordance with culture or preferences.

Unhealthful behaviors mediate approximately one-half of preventable population health deficits as well as one-half of SES-related health disparities [15]. Because behavioral and allostatic pathways interlink and reinforce one another, it is difficult to quantify their independent contribution to population health deficits. Unhealthful behaviors may reinforce allostatic drivers in a vicious cycle, as when addiction causes unemployment, which then worsens addiction. It is unknown what portion of unhealthful behaviors are induced by allostatic load, or what portion of health deficits are caused by the neuroendocrine and pro-inflammatory physiological derangements of allostasis independently of any unhealthful behaviors. Notably, SES-related health disparities are increasing even in resource-rich countries that are comparatively egalitarian and have enacted social welfare policies that are designed to blunt allostatic drivers.

## Policy Implications

Healthful behavior change is difficult because the rewards and aversions countering healthy behaviors are strong and may perpetuate unhealthy behaviors through adaptive changes in brain circuitry, particularly in environments of chronic stress. The current toolkit of nudges, education, and exhortations will likely remain inadequate. Rather, healthful behavior change may require manipulation or counteraction of the reward system so that healthful behaviors provoke less aversion and more reward, together with attention to mitigating the drivers of chronic stress. Successful examples to date are few, but include contraception, bariatric surgery, appetite suppression with medication, nicotine replacement therapy, pharmacotherapy for alcohol use disorders, and medication assisted therapy for opioid use disorder. Possible future examples include improving vaccines for reward-activating substances such as cocaine, improvements in pharmaceutical suppression of hunger, and improved methods to elevate baseline reward activation when it is impaired or intertwined with mood disorders. Because suppressing an “overactive” reward system may be difficult, an important complement is re-directing rewards toward more healthful behaviors where possible, such as by leveraging social reinforcement rewards (e.g., peer support, relational rewards), especially if they are more immediate, predictable and concrete.

Some argue that commercial determinants of health (CoDH) in general and multinational companies in particular exploit misalignment of reward response with healthful behavior, the resulting health burden from which disproportionately falls on low SES persons. CoDH are fundamental features of resource-rich environments because both originate from economic systems that provide goods and services that are desired. Further, desire that is more reward activating is more readily commercializable because it is more predictable and recurrent, and sometimes even self-escalating (e.g., colloquially, “addiction is a good business model”), whether from a rewarding substance or a rewarding internet application. Accordingly, some regulation of CoDH will always be necessary in resource rich environments. It has been observed that CoDH defend their commercial interests by exploiting power asymmetries and adopting “playbooks” [16, 17] that obscure or blatantly falsify scientific knowledge, and cartoonishly exaggerate the anti-liberty and anti-autonomy aspects of regulatory trade-offs. Discussion regarding suitable scope of CoDH regulation (e.g., proscription versus restriction versus description of harmful health effects) and principles relevant to CoDH regulatory trade-offs (e.g., for businesses; profit-making and duty to shareholders versus transparency, consumer sovereignty, and nonmalfeasance; for regulators, autonomy, liberty, and personal responsibility versus nonmalfeasance, social justice and equity) are well-described elsewhere [18].

While realigning reward system activation with population health is a different paradigm from preventing and treating disease, it is a logical extension of behavioral economics and of following determinants of health progressively upstream at population scale. If population health deficits arise from hard-to-change behaviors, and current population health approaches insufficiently change those behaviors, the field of population health should question why those behaviors are so resistant to change, and indeed, are sometimes even perpetuated. The emergence of population health as a discipline could offer a singular opportunity to incorporate fresh perspectives. Many social factors have been linked to reduced population health that have plausible allostatic mechanisms such as food insecurity, housing insecurity, clothing insecurity, energy insecurity, food deserts, and lack of greenspace. However, it is important to note that these factors are often signifiers of poor health as well as being causal mediators, and the relative importance of their causal and noncausal components are difficult to parse. For example, non-experimental data show that people live 3–5 years longer if their annual household income is $650,000 rather than $100,000 [19]. This magnitude of life expectancy increase exceeds the combined population-level effects of physical inactivity, unhealthy diet, and smoking, and dwarves the income-related health benefit seen in quasi-experimental studies, and is therefore unlikely to be mostly causal. Further underscoring the challenge of parsing causal from noncausal components of social-health associations, a systematic review of randomized trials reporting health effects of social interventions [20] yielded mostly small, inconsistent or absent health effects, even when analysis was limited to only those trials with successful social interventions and with adequate statistical power to detect meaningful changes in the health outcome(s).

However, this review together with other reports does give cause for optimism regarding the potential for improvement domains that are exceptionally intertwined with allostatic load and/or reward pathways (Figure 1), in particular the social domains of income security and early childhood development, and the health domains of mood disorders, obesity, diabetes, and vascular risk (e.g., hypertension and hyperlipidemia).

### Conclusion

The field of population health is training its sights on health shortfalls and on worsening socioeconomic health disparities. However, counteracting these problems successfully may require a perspective broader than traditionally employed by public health practitioners, in particular one that considers the power and plentitude of reward system activators in resource rich environments.

## Data Availability

The original contributions presented in the study are included in the article/supplementary material, further inquiries can be directed to the corresponding author.
